# Prevalence trends and individual patterns of ADHD medication use in pregnancy in Norway and Sweden, 2010–2019

**DOI:** 10.1007/s00228-022-03428-6

**Published:** 2022-11-29

**Authors:** Jacqueline M. Cohen, Chaitra Srinivas, Kari Furu, Carolyn E. Cesta, Johan Reutfors, Øystein Karlstad

**Affiliations:** 1 Department of Chronic Diseases, Norwegian Institute of Public Health, Oslo, Norway; 2 Centre for Fertility and Health, Norwegian Institute of Public Health, Oslo, Norway; 3 Centre for Pharmacoepidemiology, Department of Medicine, Karolinska Institutet, Stockholm, Sweden

**Keywords:** Attention deficit-hyperactivity disorder, Central nervous system stimulants, Drug utilization study, Pregnancy, Nordic health registers

## Abstract

**Purpose:**

This study aimed to describe recent trends in ADHD medication use in pregnancy in Norway and Sweden, including prevalence, individual characteristics, and patterns of use.

**Methods:**

We studied ADHD medication use (amphetamine, dexamphetamine, methylphenidate, atomoxetine, lisdexamfetamine, guanfacine) by year and age in pregnancies from 2010 to 2019 identified from the medical birth registers (gestational age ≥ 22 weeks) linked to prescribed drug registers (Norway, *N* = 577,116; Sweden, *N* = 1,118,988). We compared characteristics of those who used any ADHD medication in pregnancy to no use in pregnancy. Discontinuation was defined as no use after first trimester.

**Results:**

ADHD medication use increased from 2010 to 2019 by 3.0 users per 1000 pregnancies in Norway (from 2.5 to 5.5/1000) and by 6.3 per 1000 in Sweden (from 1.6 to 7.9/1000), mainly driven by methylphenidate and since 2015 by lisdexamfetamine. Medication use has increased among pregnant individuals of all age groups, with higher use among the youngest. Pregnant individuals who used ADHD medication were less likely to be married/cohabiting, more likely be nulliparous and to smoke. They had particularly high use of co-medication with antidepressants, anxiolytics/hypnotics, and opioids: 42% in Norway and 65% in Sweden used at least one additional class of psychotropic medication. Most individuals discontinued ADHD medication in pregnancy (85% Norway, 78% Sweden).

**Conclusion:**

ADHD medication use during pregnancy increased in Norway and Sweden in the last decade. However, discontinuation rates during pregnancy were high. Those who used ADHD medication had more risk factors for pregnancy complications including low parity, smoking, and other psychotropic drug use.

**Supplementary Information:**

The online version contains supplementary material available at 10.1007/s00228-022-03428-6.

## Introduction

Attention-deficit hyperactivity disorder (ADHD) is defined by symptoms of inattention and hyperactivity-impulsivity that arise in childhood and interfere with social, academic, or occupational functioning [1, 2]. In the last two decades, there has been increasing awareness that the disorder often persists into adulthood [3]. Population surveys suggest a prevalence of 1–8% among adults [4, 5]. Adult ADHD is associated with high prevalence of comorbid mood, anxiety, and substance use disorders [6, 7]. The incidence and prevalence of ADHD diagnosis among adults has increased in various populations in recent years [8–10], translating to increases in adult use of ADHD medication, primarily methylphenidate, and amphetamines, in the last decade [11, 12]. Increasing ADHD medication use has been substantial among females of reproductive age [10, 13]. In the UK from 2000 to 2015, use increased annually by 1.3% per year for females aged 19–24 and by 1.2% per year for females aged 24–49 [10]. In the USA from 2003 to 2015, use among females 15–44 increased from approximately 1% to 4%, but with increases more than five-fold for those aged 25–34 [13]. Consequently, use during pregnancy was shown to increase dramatically in the USA and Denmark, starting around 2005 [14, 15]. In a US case–control study of birth defects, 0.2% of study participants used ADHD medication in pregnancies conceived before 2006, and this rose to 1.3% in 2013 [14]. A roughly six-fold increase in use during pregnancy was also observed in Denmark between 2006 and 2010 [15]. However, knowledge about more recent patterns of use in pregnancy is lacking. It is important for clinicians, others who provide information to pregnant and lactating individuals (e.g., teratology information services), and policy-makers to be aware of how medication use in pregnancy has changed in recent years. If use during pregnancy is increasing, it would reflect a need for greater consideration of pregnancy in treatment decisions and discussion of pregnancy planning with patients. For researchers, understanding recent trends illuminates which medications are most important to have safety data on.

### Aims of the study

The objectives of this study were to describe recent trends in ADHD medication use in pregnancy in Norway and Sweden, including prevalence of use, characteristics of users, and patterns of use.

## Material and methods

We used data from the Medical Birth Registry of Norway (MBRN) linked to the Norwegian Prescription Database (NorPD), and the Swedish Medical Birth Register (SMBR) linked to the Prescribed Drug Register (PDR). Both countries have virtually complete registration of births from 22 weeks with near universal second trimester ultrasound to confirm gestational age. Therefore, we included all births (singletons, multiples, live born, and stillborn) from 22 weeks. The start of pregnancy (first day of the last menstrual period, LMP) was calculated by subtracting the gestational age at birth in days from the delivery date. To ensure that the date of the start of pregnancy was as accurate as possible, we excluded births with a gestational age > 44 weeks, or < 35 weeks with an implausibly high recorded birthweight > 4 standard deviations from the mean for gestational age and sex [16].

We studied ADHD medication use in pregnant individuals who gave birth from 2010 to 2019 (Norway, *N* = 577,116; Sweden, *N* = 1,118,988, Supplementary Material 1). In this paper, we use the inclusive term “pregnant individuals” that acknowledges the existence of transgender and nonbinary people in addition to those who identify as women in the birth registers. Population-based register data includes information about sex assigned at birth (male or female), rather than gender identity. The following ADHD drugs of interest were identified by Anatomic Therapeutic Chemical (ATC) codes: amphetamine (ATC N06BA01) dexamphetamine (N06BA02), methylphenidate (N06BA04), atomoxetine (N06BA09), lisdexamfetamine (N06BA12), and guanfacine (C02AC02). We defined pregnancy use as at least one prescription filled from 90 days before LMP to delivery. Since a three-month supply is generally dispensed for reimbursed drugs in Norway and Sweden, this definition captures all potential use in pregnancy but will also include some who discontinued drug treatment before the start of pregnancy. However, because several ADHD drugs are classified as narcotics in Norway and Sweden, doctors may conservatively prescribe them for less than three months at a time. The registers capture all prescribed drugs dispensed to outpatients, regardless of whether they are prescribed by general practitioners or specialists, or whether they are reimbursed. ADHD medication should only be prescribed in Norway and Sweden following a diagnosis from a relevant specialist. ADHD medications can only be prescribed by specialists in Sweden (except for atomoxetine and guanfacine which any doctor may prescribe), while in Norway, general practitioners can be responsible for prescribing to patients after initiation by a specialist. There are reimbursement restrictions requiring that methylphenidate be used before other ADHD medications, except when contraindicated.

We described the trends in prevalence of use of ADHD medication per 1000 pregnancies from 2010 to 2019, overall, by drug, and by age group. We also compared overall ADHD medication use in pregnancy to use in the general population by extracting the prevalence of use per 1000 females in the population by age group from publicly available statistics (Norway: www.norpd.no, Sweden: sdb.socialstyrelsen.se/if_lak/val.aspx).

We described the characteristics of pregnant individuals who used ADHD medication versus pregnant individuals without use. Characteristics included age, parity, marital status, tobacco smoking at the start of pregnancy, and co-medication with other psychotropic drugs (from LMP-90 days to delivery): antidepressants (N06A), antiepileptics (N03A), antipsychotics (N05A), anxiolytics, hypnotics and sedatives (N05B, N05C), and opioids (N02A).

Among those with ADHD medication use in pregnancy, we described individual patterns of use by prescription fills for the following periods: 3 months before pregnancy (PRE: LMP-90 to LMP-1), first trimester (T1: LMP to LMP + 97 days), second trimester (T2: LMP + 98 to LMP + 202 days), and third trimester (T3: LMP + 203 days to delivery). We defined discontinuation as prescription fills early (PRE or T1) but not later in pregnancy (T2 or T3), continuous use as fills early (PRE or T1) and late in pregnancy (T2 or T3), and initiation as fills only after first trimester.

In addition to the individual patterns of use described above, we described the timing of ADHD medication use in relation to pregnancy. We calculated the prevalence of use from 6 months before to six months after pregnancy. Then, we compared the prevalence in each period to use in the three months before pregnancy.

## Results

**Fig. 1:**
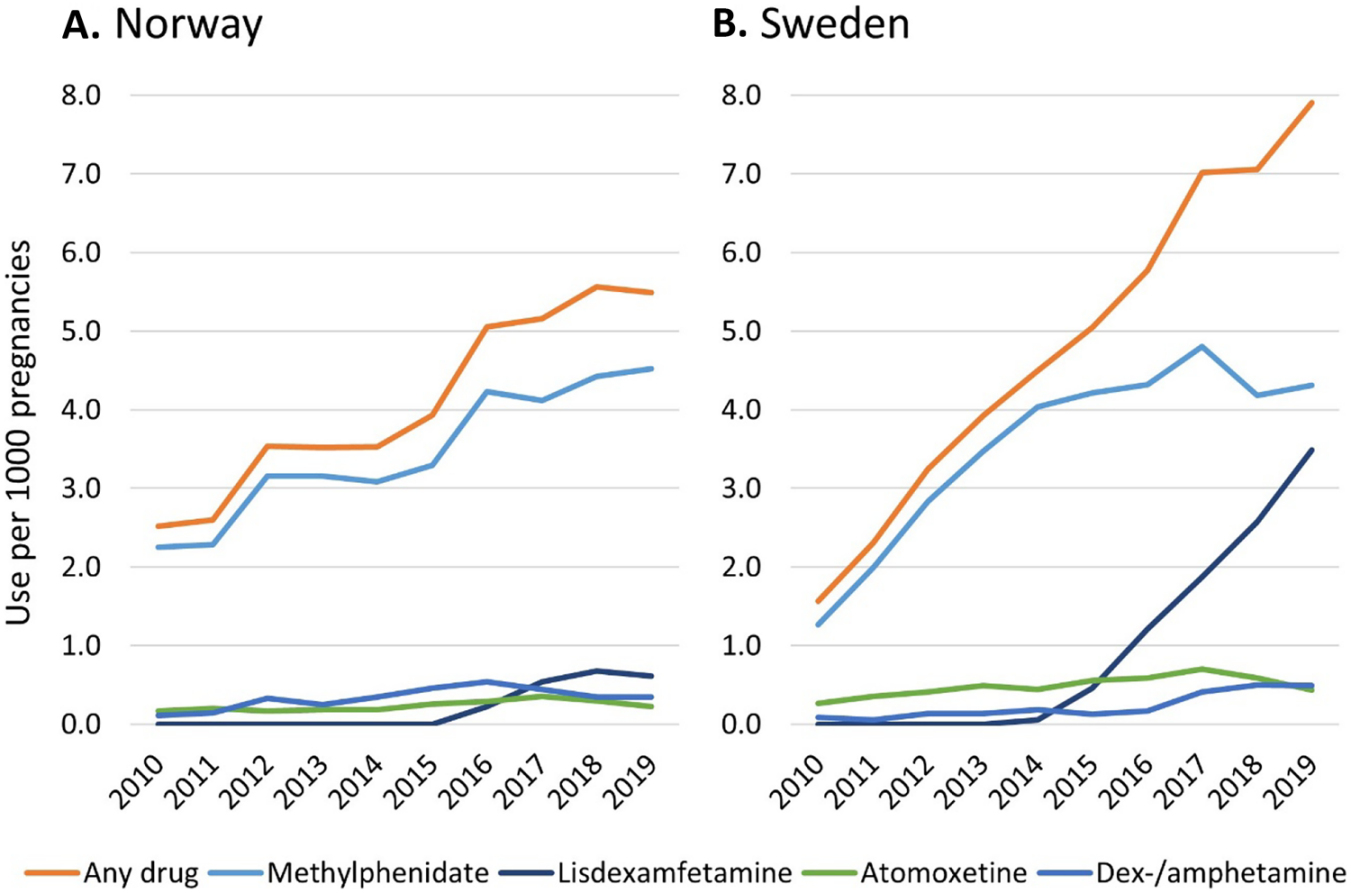
ADHD medication use per 1000 pregnancies from 2010 to 2019 overall and by drug in Norway and Sweden**.** Dexamphetamine and amphetamine use were added together so a few pregnancies were counted twice (< 5 in each country). Guanfacine is not shown because the number of users was too small

ADHD medication use increased by 3.0 per 1000 pregnancies (from 2.5 to 5.5/1000) in Norway and by 6.3 per 1000 (from 1.6 to 7.9/1000) in Sweden from 2010 to 2019 (Fig. 1). In both countries, increase in ADHD medication use was mainly driven by methylphenidate and since 2015 by lisdexamfetamine in Sweden. In 2019, lisdexamfetamine accounted for almost half of ADHD medication use in pregnancy in Sweden, while it was closer to 10% of use in Norway.

**Fig. 2:**
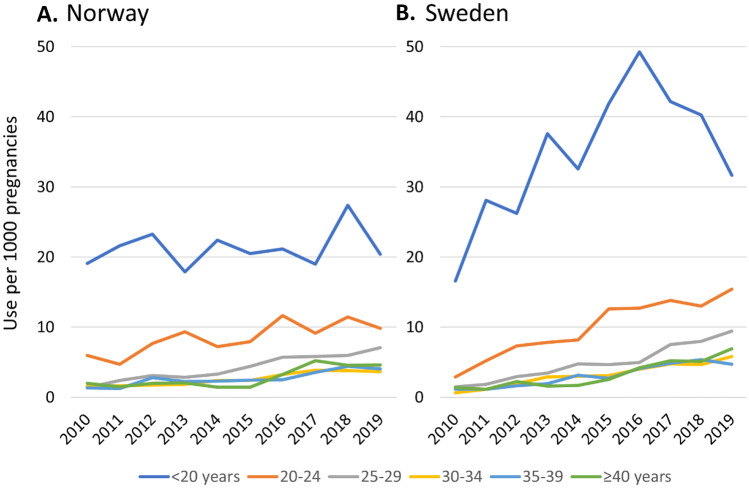
ADHD medication use per 1000 pregnancies by age at birth in Norway and Sweden. ADHD medication includes amphetamine, dexamphetamine, methylphenidate, atomoxetine, lisdexamfetamine, and guanfacine. To maintain anonymity in the data, we have replaced counts of < 3 individuals with 3

ADHD medication use among females in the general population rose continuously in all age groups from 2010 to 2019 in Norway and Sweden (Supplementary Material 2). Similarly, medication use in pregnancy has increased in all age groups in Norway and Sweden (Fig. 2). However, in most age groups, less ADHD medication was used during pregnancy except for individuals who gave birth < 20 years of age who used more until the most recent years in both countries. Individuals who gave birth before age 20 were much more likely to use ADHD medication. Use among the youngest pregnant individuals in Norway was more stable, whereas it roughly doubled in Sweden during the same period. However, use among the youngest pregnant individuals in Sweden peaked in 2016 and declined thereafter.

From 2010 to 2019, ADHD medication was used in 2339 (0.41%) of 577,116 pregnancies in Norway and 5436 (0.49%) of 1,118,988 pregnancies in Sweden. Pregnant individuals using ADHD medication were more often younger, less likely to have had a previous birth, or be married or cohabiting with a partner, and more likely to smoke (Table 1). Individuals who used ADHD medication during pregnancy were much more likely to use other psychotropic medication compared to those not using ADHD medication; 42% in Norway and 65% in Sweden used at least one other class of psychotropic medication during the same period (compared to 11% and 12%, for those with no ADHD medication use, respectively). The most frequently used co-medications were anxiolytics, hypnotics, and sedatives, with co-medication almost twice as high in Sweden as Norway (40% vs. 20%), while use in those without ADHD medication was more similar (4% vs. 3%). Antidepressant use was more than twice as likely among those with ADHD medication use in Sweden versus Norway (38% vs. 16%), and the use in those without ADHD medication was also about twice as high (6% vs. 3%). Opioid use in pregnancy was similar in those with and without ADHD medication use in both countries, with use about three times higher in those who used ADHD medication.

**Table 1:** Characteristics of individuals giving birth from 2010 to 2019 in Norway and Sweden stratified by ADHD medication use

	**Norway**		**Sweden**	
	**ADHD medication use (*N*** **= 2339)**	**No ADHD medication use (*N*** **= 574,777)**	**ADHD medication use (*N*** **= 5436)**	**No ADHD medication use (*N*** **= 1,113,554)**
Age at birth^a^				
< 20	160 (6.8)	7468 (1.3)	421 (7.7)	12,262 (1.1)
20–29	1349 (57.7)	254,397 (44.3)	3010 (55.4)	475,415 (42.7)
30–39	774 (33.1)	292,471 (50.9)	1851 (34.1)	579,132 (52.0)
≥ 40	56 (2.4)	20,441 (3.6)	154 (2.8)	46,743 (4.2)
Parity				
Nulliparous	1180 (50.4)	243,356 (42.3)	2851 (52.4)	483,137 (43.4)
Primiparous	703 (30.1)	211,872 (36.9)	1435 (26.4)	414,399 (37.2)
Multiparous	456 (19.5)	119,549 (20.8)	1150 (21.2)	216,018 (19.4)
Marital status				
Married/cohabiting	1805 (77.2)	537,329 (93.5)	3776 (69.5)	990,939 (89.0)
Other	502 (21.5)	31,397 (5.5)	1660 (30.5)	122,615 (11.0)
Missing	32 (1.4)	6051 (1.1)	0	0
Smoking in early pregnancy				
Yes	551 (23.6)	33,741 (5.9)	1397 (25.7)	54,488 (4.9)
No	1553 (66.4)	474,611 (82.6)	3826 (70.4)	1,022,809 (91.9)
Missing	235 (10.0)	66,425 (11.6)	213 (3.9)	36,257 (3.3)
Co-medication				
Antidepressants	363 (15.5)	15,317 (2.7)	2074 (38.2)	62,468 (5.6)
Antiepileptics	118 (5.0)	4300 (0.7)	588 (10.8)	8207 (0.7)
Antipsychotics	181 (7.7)	6625 (1.2)	508 (9.3)	4809 (0.4)
Anxiolytics, hypnotics, and sedatives	474 (20.3)	15,788 (2.7)	2170 (39.9)	40,356 (3.6)
Opioids	390 (16.7)	33,272 (5.8)	949 (17.5)	61,044 (5.5)
> 1 of the above	374 (16.0)	10,645 (1.9)	1827 (33.6)	30,330 (2.7)
0 of the above	1353 (57.8)	512,693 (89.2)	1915 (35.2)	974,898 (87.5)

^a^*n* = 2 missing age at birth in Sweden

Methylphenidate was the predominately used ADHD medication in both countries with a prevalence > 3 per 1000 pregnancies in Norway and Sweden (Table 2). This was followed by dexamphetamine in Norway (0.3/1000) and by lisdexamfetamine (1.0/1000) in Sweden. Over 90% used only one drug in the pregnancy period. More used two or more drug types in Sweden than in Norway. Most people discontinued ADHD medication use during pregnancy in both countries, with continuation slightly higher in Sweden (21% vs. 14%). About 1% initiated treatment after the first trimester. Among those who discontinued ADHD medication, 36% (712/1980) in Norway and 35% (1494/4221) in Sweden re-initiated in the 6 months following pregnancy.

**Table 2:** Description of use of ADHD medication in pregnancy

	**Norway** ***N*** **= 2339**	**Sweden** ***N*** **= 5436**
Use of specific ADHD medications, *N* (per 1000 pregnancies)		
Methylphenidate	1977 (3.43)	3977 (3.55)
Dexamphetamine	174 (0.30)	220 (0.20)
Atomoxetine	134 (0.23)	543 (0.49)
Lisdexamfetamine	113 (0.15)	1095 (0.98)
Amphetamine	28 (0.05)	37 (0.03)
Guanfacine	< 3	14 (0.01)
Number of ADHD medications used in pregnancy, *N* (%)		
1	2257 (96.5)	5011 (92.2)
2	76 (3.2)	401 (7.4)
3 +	6 (0.3)	24 (0.4)
Pattern of ADHD medication use in pregnancy, *N* (%)		
Discontinued	1980 (84.7)	4221 (77.6)
Continued	337 (14.4)	1140 (21.0)
Initiated	20 (0.9)	68 (1.3)

We also investigated which drugs predominated use in pregnancy at different ages. Older pregnant individuals were more likely to use dexamphetamine or amphetamine. Atomoxetine use was highest in pregnant individuals < 20 years in both countries. Methylphenidate use was highest among pregnant individuals 20–29 in Norway and those < 20 and ≥ 40 in Sweden. Lisdexamphetamine use was highest in pregnant individuals 30–39 in Norway and used in a similar proportion of pregnancies in all age groups ≥ 20 years in Sweden (Supplementary Material 3).

**Fig. 3:**
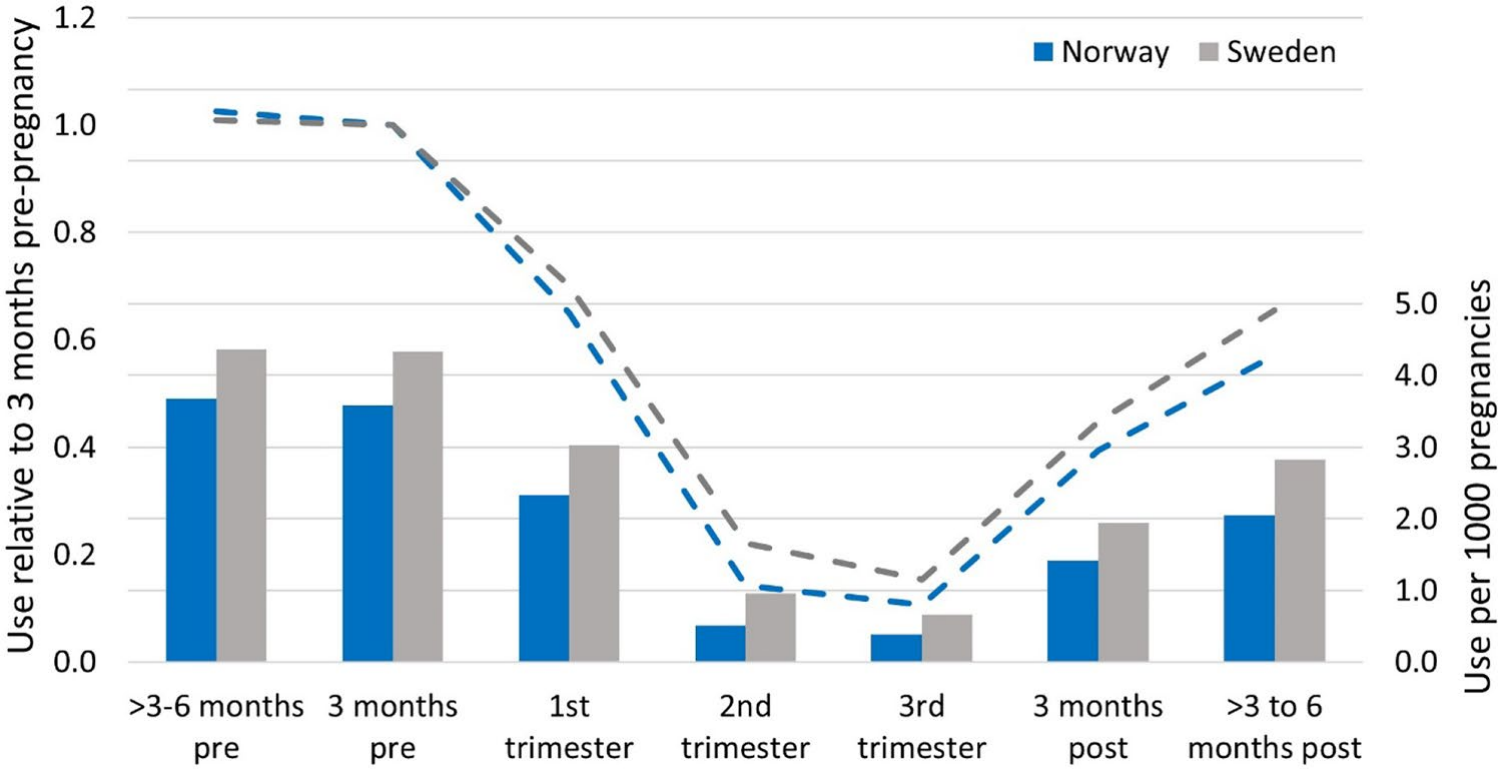
Timing of ADHD medication use before, during, and after pregnancy in Norway and Sweden. The bars represent the prevalence (per 1000 pregnancies) of ADHD medication use in each time-period, and the dashed lines represent the ratio of the prevalence of ADHD medication use in each time-period, relative to the 3 months before pregnancy. ADHD medication includes amphetamine, dexamphetamine, methylphenidate, atomoxetine, lisdexamfetamine, and guanfacine

When we looked at the timing of ADHD medication use before, during, and after pregnancy in Norway and Sweden, we observed that the prevalence of use was similar during the period from three to 6 months before pregnancy as the 3 months before pregnancy (Fig. 3). The trends in the timing of use were similar in Norway and Sweden. However, overall use from 2010 to 2019 was lower in Norway.

## Discussion

ADHD medication use during pregnancy increased in Norway and Sweden between 2010 and 2019, predominantly methylphenidate but with a notable recent increase in lisdexamfetamine in Sweden. At the start of the decade, use in pregnancy was higher in Norway, but use rose more steeply in Sweden and by 2019 was more prevalent in Sweden. However, discontinuation rates during pregnancy were high (85% in Norway, 78% in Sweden), and only around one-third of those individuals re-initiated in the 6 months after giving birth.

The increase in ADHD medication use in pregnancy in Norway was only seen in individuals over 20 years of age. Diagnosis of ADHD was stable for female children ages 6–17 years in Norway between 2008 and 2016 [17]. In Sweden, ADHD medication use in pregnancy increased in all age groups. Diagnosis of ADHD in both children and adults increased in Sweden in the early part of the study period [18, 19]. There were also guidelines for the diagnosis and treatment of ADHD that included management of ADHD in adults published in Norway in 2014 and in Sweden in 2015 and 2016 that reflected changes in recognition and treatment of adults [20–22], none of which provided recommendations for pregnant individuals. However, published data from Norway and Sweden on incident diagnoses among adults for the most recent years are lacking.

Lisdexamfetamine was marketed from September 2013 in Sweden and was approved for adults in 2015 [23]. In Norway, it was marketed from September 2014 and approved and marketed for adults since May 2018. Lisdexamfetamine is eligible for reimbursement in Norway and Sweden if the response to previous methylphenidate therapy is not considered clinically sufficient [24, 25]. Lisdexamfetamine is a prodrug and therefore may have lower potential for abuse than other stimulant ADHD medications [26]. Earlier approval for adults in Sweden and lower abuse potential may have played a role in the rapid increase in use in Sweden, but it is unclear why the use is so much higher than in Norway.

Data on ADHD medication safety in pregnancy is limited, especially for drugs other than methylphenidate [27]. There is evidence that methylphenidate is associated with a modest increase in risk of major cardiac malformations (risk ratio 1.28) [28]. ADHD medication use has also been linked to preeclampsia and preterm birth, but these studies may have had residual confounding [29, 30]. The current study shows that individuals who use ADHD medication in pregnancy may have a higher prevalence of risk factors for pregnancy complications due to their younger age, nulliparity, smoking habits, and use of other psychotropic medications which are important to account for to reduce confounding bias in drug safety studies.

We could not include all pregnancies in this study, only those resulting in a birth. Some research has shown that pregnant individuals with ADHD are at increased risk of miscarriage, and whether this is driven by the underlying condition or medication for ADHD is not well understood [31, 32]. Pregnant individuals with ADHD are also more likely to have an induced abortion [15]. Therefore, actual ADHD medication use in pregnancy may be underestimated since we did not include miscarriages and induced abortions.

Another limitation of our study is that we could only study prescriptions filled and do not know when the drugs were actually taken. Previous research has shown that prescription fills for ADHD medication decline rapidly in the weeks after a pregnancy may be recognized [33]. Therefore, many of the individuals who are considered to use ADHD medication during pregnancy may only do so for a very limited period. Some of those who filled a prescription in the 90 days before pregnancy may not have taken it during pregnancy at all. Over one-third of those with pregnancy use in this study only had a prescription in the 90 days before pregnancy. Therefore, we likely overestimate actual use in pregnancy. However, our aim was to identify all individuals with potential ADHD medication use in pregnancy. A study that is concerned with understanding the risk of specific adverse outcomes associated with ADHD medication use should likely have a stricter definition to define pregnancy exposure [34].

In conclusion, ADHD medication use in pregnant individuals has increased in both Norway and Sweden across all age groups. However, discontinuation rates are high. Most of the increase is driven by increasing use of methylphenidate, but in Sweden, the approval of lisdexamfetamine for adults in 2015 seems to drive the most recent trend. Those who used ADHD medication had more risk factors for pregnancy complications including low parity, smoking, and other psychotropic drug use, which is important to account for in future drug safety studies. Future studies should focus on the safety of lisdexamfetamine and also on the consequences of discontinuing ADHD medication for mental health in pregnancy and postpartum.

## Supplementary Information

Below is the link to the electronic supplementary material.

Supplementary file1 (PDF 192 KB)

Supplementary file2 (PDF 363 KB)

Supplementary file3 (PDF 177 KB)

## Data Availability

The data used for this study come from the health registers of Norway and Sweden and are available to other researchers upon ethical approval and application to the register holders. The authors may not share the study data due to regulations which restrict access and distribution to those with ethical and legal permission to use the data.
