# Supplementary material for: Prevalence trends and individual patterns of ADHD medication use in pregnancy in Norway and Sweden, 2010–2019
Source: Eur J Clin Pharmacol. 2022 Nov 29;79(1):173–80. doi: 10.1007/s00228-022-03428-6 (PMC9816174; doi:10.1007/s00228-022-03428-6)
Supplement: Supplementary file 1 — Supplementary file1 (PDF 192 KB) [file 228_2022_3428_MOESM1_ESM.pdf]

European Journal of Clinical Pharmacology

Supplementary Material 1

Prevalence trends and individual patterns of ADHD medication use in pregnancy in Norway and Sweden, 2010-2019

Jacqueline M. Cohen,[1,2] Chaitra Srinivas,[1,2] Kari Furu,[1,2] Carolyn E. Cesta,[3] Johan Reutfors,[3] Øystein Karlstad[1]

[1] Department of Chronic Diseases, Norwegian Institute of Public Health, Oslo, Norway

[2] Centre for Fertility and Health, Norwegian Institute of Public Health, Oslo, Norway

[3] Centre for Pharmacoepidemiology, Department of Medicine, Karolinska Institutet, Stockholm, Sweden

Corresponding author: Jacqueline M. Cohen, PhD, Senior Researcher, Norwegian Institute of Public Health, jacqueline.cohen@fhi.no

Table S1: Number of pregnancies per year by age at birth in Norway, 2010-2019

	Age at birth						
	**<20**	**20-24**	**25-29**	**30-34**	**35-39**	**≥40**	**Total**
**2010**	1,309	9,036	18,819	19,704	9,893	1,985	60,746
**2011**	1,111	8,673	18,707	19,328	9,777	2,004	59,600
**2012**	945	8,607	18,802	19,592	9,675	1,984	59,605
**2013**	894	8,023	18,624	19,495	9,304	1,964	58,304
**2014**	803	7,458	18,720	19,813	9,555	2,091	58,440
**2015**	732	7,061	19,118	19,549	9,731	2,072	58,263
**2016**	615	6,515	19,070	20,156	9,627	2,143	58,126
**2017**	474	5,706	18,461	19,759	9,336	2,107	55,843
**2018**	402	5,325	17,544	19,937	9,264	1,983	54,455
**2019**	343	4,686	16,791	20,164	9,586	2,164	53,734
**Total**	7628	71090	184656	197497	95748	20,497	577116

Table S2: Number of pregnancies per year by age at birth in Sweden, 2010-2019

	Age at birth						
	**<20**	**20-24**	**25-29**	**30-34**	**35-39**	**≥40**	**Total**
**2010**	1,751	14,860	32,174	37,979	20,906	4,360	112,030
**2011**	1,674	14,553	31,148	36,498	20,153	4,386	108,412
**2012**	1,449	14,730	31,858	37,069	19,824	4,498	109,428
**2013**	1,356	14,454	32,587	37,427	19,554	4,466	109,844
**2014**	1,259	14,208	34,591	37,711	19,462	4,760	111,991
**2015**	1,098	13,810	35,818	37,502	19,575	4,696	112,499
**2016**	1,076	13,201	37,215	38,247	20,398	4,975	115,112
**2017**	1,067	12,146	36,542	38,283	20,158	4,978	113,174
**2018**	1,068	11,551	36,844	39,554	20,183	4,717	113,917
**2019**	885	10,509	35,626	40,166	20,334	5,061	112,581
**Total**	12683	134022	344403	380436	200,547	46,897	1,118,988
