# Supplementary material for: Prevalence trends and individual patterns of ADHD medication use in pregnancy in Norway and Sweden, 2010–2019
Source: Eur J Clin Pharmacol. 2022 Nov 29;79(1):173–80. doi: 10.1007/s00228-022-03428-6 (PMC9816174; doi:10.1007/s00228-022-03428-6)

European Journal of Clinical Pharmacology

Supplementary Material 2

Prevalence trends and individual patterns of ADHD medication use in pregnancy in Norway and Sweden, 2010-2019

Jacqueline M. Cohen,[1,2] Chaitra Srinivas,[1,2] Kari Furu,[1,2] Carolyn E. Cesta,[3] Johan Reutfors,[3] Øystein Karlstad[1]

[1] Department of Chronic Diseases, Norwegian Institute of Public Health, Oslo, Norway

[2] Centre for Fertility and Health, Norwegian Institute of Public Health, Oslo, Norway

[3] Centre for Pharmacoepidemiology, Department of Medicine, Karolinska Institutet, Stockholm, Sweden

Corresponding author: Jacqueline M. Cohen, PhD, Senior Researcher, Norwegian Institute of Public Health, jacqueline.cohen@fhi.no

Figure S1. Comparison of any ADHD medication use per 1000 pregnancies (left) with use per 1000 female individuals in the general population (right), by age group. ADHD medication includes amphetamine, dexamphetamine, methylphenidate, atomoxetine, lisdexamfetamine, and guanfacine. Data sources: [www.norpd.no (Norway)](www.norpd.no), [sdb.socialstyrelsen.se/if_lak/val.aspx (Sweden)](sdb.socialstyrelsen.se/if_lak/val.aspx)

A. Norway

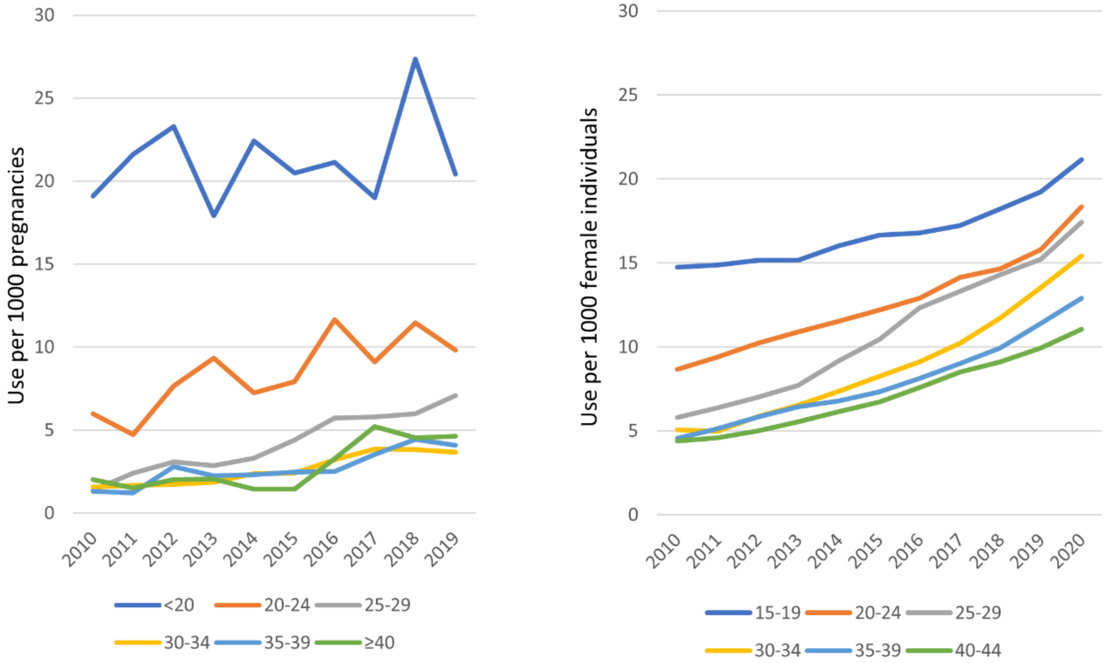

B. Sweden

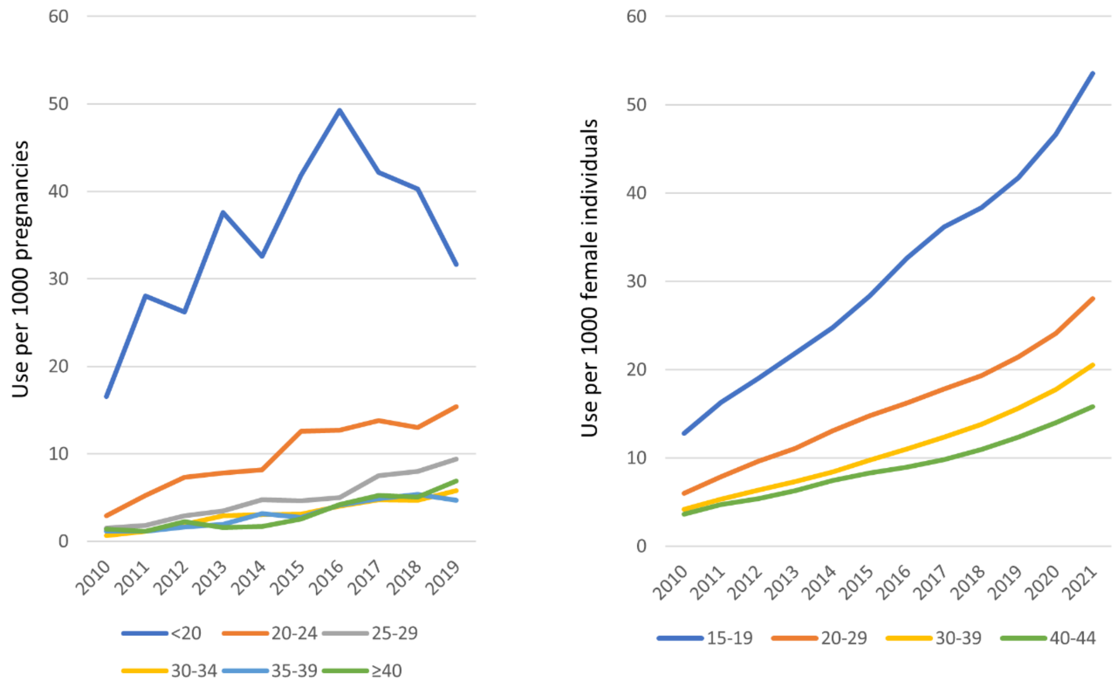

Supplement: Supplementary file 2 — Supplementary file2 (PDF 363 KB) [file 228_2022_3428_MOESM2_ESM.pdf]
