# Supplementary material for: Prevalence trends and individual patterns of ADHD medication use in pregnancy in Norway and Sweden, 2010–2019
Source: Eur J Clin Pharmacol. 2022 Nov 29;79(1):173–80. doi: 10.1007/s00228-022-03428-6 (PMC9816174; doi:10.1007/s00228-022-03428-6)
Supplement: Supplementary file 3 — Supplementary file3 (PDF 177 KB) [file 228_2022_3428_MOESM3_ESM.pdf]

European Journal of Clinical Pharmacology

Supplementary Material 3

Prevalence trends and individual patterns of ADHD medication use in pregnancy in Norway and Sweden, 2010-2019

Jacqueline M. Cohen,[1,2] Chaitra Srinivas,[1,2] Kari Furu,[1,2] Carolyn E. Cesta,[3] Johan Reutfors,[3] Øystein Karlstad[1]

[1] Department of Chronic Diseases, Norwegian Institute of Public Health, Oslo, Norway

[2] Centre for Fertility and Health, Norwegian Institute of Public Health, Oslo, Norway

[3] Centre for Pharmacoepidemiology, Department of Medicine, Karolinska Institutet, Stockholm, Sweden

Corresponding author: Jacqueline M. Cohen, PhD, Senior Researcher, Norwegian Institute of Public Health, jacqueline.cohen@fhi.no

Table S3. Number of users and proportion of all ADHD medication use in pregnancy that includes each of the specific medications in total and by age at birth

N (%)†	All ADHD Medications	Methyl-phenidate	Atomoxetine	Lisdexamfet-amine	Dex-/amphet-amine
**Norway**					
Total	2339 (100)	1977 (85)	134 (6)	113 (5)	202 (9)
<20	160 (100)	135 (84)	17 (11)	7 (4)	4 (3)
20-29	1349 (100)	1163 (86)	80 (6)	48 (4)	96 (7)
30-39	774 (100)	634 (82)	33 (4)	<58 (7)	95 (12)
≥40	56 (100)	45 (80)	4 (7)	<3	7 (13)
**Sweden**					
Total	5436 (100)	3977 (73)	543 (10)	1095 (20)	257 (5)
<20	421 (100)	327 (78)	75 (18)	58 (14)	0
20-29	3010 (100)	2231 (74)	305 (10)	590 (20)	101 (3)
30-39	1851 (100)	1301 (70)	154 (8)	415 (22)	145 (8)
≥40	154 (100)	118 (77)	9 (6)	32 (21)	11 (7)

† Percent values across the rows sum to >100 since some pregnant individuals used more than one medication during the pregnancy period. Dexamphetamine and amphetamine use were added together so a few pregnancies were counted twice (<5 in each country). Use of guanfacine (not shown) was rare, <1% in all age groups in both countries.
